# Rheumatoid arthritis and mitochondrial homeostasis: The crossroads of metabolism and immunity

**DOI:** 10.3389/fmed.2022.1017650

**Published:** 2022-09-23

**Authors:** Liu Cui, Jing Weiyao, Su Chenghong, Liu Limei, Zhang Xinghua, Yuan Bo, Du Xiaozheng, Wang Haidong

**Affiliations:** ^1^College of Acupuncture-Moxibustion and Tuina, Gansu University of Chinese Medicine, Lanzhou, China; ^2^Acupuncture and Moxibustion Department, Gansu Provincial Hospital of Traditional Chinese Medicine (TCM), Lanzhou, China; ^3^Acupuncture and Pain Department, Affiliated Hospital of Gansu University of Traditional Chinese Medicine (TCM), Lanzhou, China; ^4^Rheumatoid Bone Disease Center, Gansu Provincial Hospital of Traditional Chinese Medicine (TCM), Lanzhou, China

**Keywords:** rheumatoid arthritis, mitochondrial homeostasis, energy metabolism, immune cells, apoptosis and proliferation, inflammatory pathways

## Abstract

Rheumatoid arthritis is an autoimmune disease characterized by chronic symmetric synovial inflammation and erosive bone destruction. Mitochondria are the main site of cellular energy supply and play a key role in the process of energy metabolism. They possess certain self-regulatory and repair capabilities. Mitochondria maintain relative stability in number, morphology, and spatial structure through biological processes, such as biogenesis, fission, fusion, and autophagy, which are collectively called mitochondrial homeostasis. An imbalance in the mitochondrial homeostatic environment will affect immune cell energy metabolism, synovial cell proliferation, apoptosis, and inflammatory signaling. These biological processes are involved in the onset and development of rheumatoid arthritis. In this review, we found that in rheumatoid arthritis, abnormal mitochondrial homeostasis can mediate various immune cell metabolic disorders, and the reprogramming of immune cell metabolism is closely related to their inflammatory activation. In turn, mitochondrial damage and homeostatic imbalance can lead to mtDNA leakage and increased mtROS production. mtDNA and mtROS are active substances mediating multiple inflammatory pathways. Several rheumatoid arthritis therapeutic agents regulate mitochondrial homeostasis and repair mitochondrial damage. Therefore, modulation of mitochondrial homeostasis would be one of the most attractive targets for the treatment of rheumatoid arthritis.

## Introduction

Rheumatoid arthritis (RA) is an autoimmune disease characterized by chronic symmetric synovial inflammation and erosive bone destruction. It mainly involves small joints of the limbs and results in painful swelling, stiffness, and even deformity of the joints, as well as cardiovascular, interstitial lung, connective tissue, and other systemic lesions (1). The global prevalence of RA is ~0.5–1.0%, and the incidence is twice as high in women as in men. The age of onset differs at various stages, but it most commonly occurs around the age of 50 years. RA remains incurable, with a disability rate of 61.3%, due to destruction of articular cartilage, bone, and joint capsule, which is why it is called “the cancer that never dies.” It increases the burden on patients' mental health, family, and social economy (2, 3). Current studies have shown that abnormal energy metabolism, RA-FLS (fibroblastic synovial cells) pathological behavior, and joint inflammatory response environment are the key factors that mediate RA (4). FLS is the main cell that constitutes synovial tissue. It is highly invasive upon abnormal activation, which can mediate inflammation and joint destruction, while the inflammatory micro-environment is highly activated by the RA-FLS glycolytic pathway and altered metabolism of key macromolecules, such as amino acids, glucose, and lipids. These changes in energy metabolism are involved in abnormal FLS activation and synovial inflammation (5). The increase in glycolytic and amino acid metabolites is followed by a disturbance in primitive T cell metabolism, enhanced activity of the pentose phosphate pathway, and a decrease in intracellular reactive oxygen species (ROS) levels, which promotes Th1 and Th17 differentiation, glucose uptake, and increased glycolytic flux to provide FLS cells with the required energy (6). The current clinical treatment of RA mostly uses the combination of non-steroidal anti-inflammatory drugs (NSAIDs) and disease-modifying anti-rheumatic drugs (DMARDs) with the main aim of controlling the disease and alleviating the inflammatory response. However, the current therapeutic effect of RA is still unsatisfactory, and the regulation of energy metabolism can be a potential target for the treatment of RA (7).

Mitochondria are semi-autonomous organelles with bilayer membrane structures, consisting of an outer membrane, a membrane gap, and an inner membrane. They are the “powerhouse” of eukaryotic cells, producing ATP through the tricarboxylic acid cycle and oxidative phosphorylation (OXPHOS) to provide energy for the cell (8). Additionally, mitochondria are involved in various biological processes, such as signal transduction, reduction-oxidation, cell cycle regulation, and apoptosis. Mitochondrial homeostasis refers to the maintenance of the relative stability of the number, morphology, and spatial structure of mitochondria in all organs of the body through biological processes, such as biogenesis, fission, fusion, and autophagy, to ensure the overall energy metabolic requirements of the body. The imbalance of mitochondria homeostasis can lead to the development of autoimmune rheumatic diseases, including scleroderma, dry syndrome and systemic lupus erythematosus (SLE), etc. (9–11). SLE is characterized by an imbalance in T-cell mitochondrial homeostasis, mainly involving enhanced of mitochondrial biosynthesis and inhibition of autophagy (12, 13). In recent years, several studies have shown that the imbalance of the endostatic environment due to mitochondrial impairment plays a key role in the pathology of RA and is one of the central aspects of RA pathology (14). Monocytes from patients with RA show downregulation of several mitochondrial proteins, increase in mtDNA copy number, and low expression of mitochondrial membrane potential, superoxides, and ATPs. This suggests that mitochondria are damaged, and alterations in mtDNA replication and transcription mediate the downstream oxidative stress response, promoting the secretion of inflammatory factors and exacerbating the inflammatory response in RA (15). Additionally, the proliferation of RA synovial tissue vasculature leads to an increased demand for mitochondrial electron transfer energy, which is aggravated by the presence of single nucleotide variant (SNV) aggregation on mitochondrial respiratory chain-associated proteins. This results in increased electron leakage and reduction of oxygen molecules to superoxide anions to generate ROS, further promoting glycolysis and inflammatory responses (16). Meanwhile, microvascular disorders in RA synovial tissues and local presence of a hypoxic environment in cells can induce the production of ROS and reactive nitrogen species, which can mediate the release of inflammatory factors from NF-κB, STAT3, and other pathways. Eventually, this aggravates inflammatory response in RA and promotes apoptosis of chondrocytes (17). Therefore, the abnormal state of mitochondria in patients with RA is closely related to RA synovial inflammation and abnormal energy metabolism.

The pathogenesis of RA is complex, and in addition to abnormal energy metabolism due to mitochondrial damage, factors such as sex hormones, environment, smoking and immune response also play an important role (18). Studies have reported that women are more prone to rheumatoid arthritis and have higher disability, which can be attributed to differences in sex hormone levels (19). Androgen levels are negatively correlated with RA severity and have an immunosuppressive function (20, 21), while oestrogens enhance humoral immunity and have a promotive effect on immune activity (22). Whereas, both estrogens and androgens interfere with processes such as mitochondrial oxidative phosphorylation, mitochondrial biogenesis and mitochondrial autophagy (23), there is a lack of research on the effects in RA. Smoking can increases the risk of anti-CCP antibody positivity and RF formation, which are important factors in the development of RA (24). The particulate tar in cigarette contains high concentrations of free radicals that can induce oxidative stress (25). Nicotine exposure also induces oxidative stress, which increases mitochondrial membrane permeability and mtDNA leakage, mediating synovial inflammation (26). The activity of the major histocompatibility complex (MHC) and anti-citrullinated protein anti-bodies (ACPAs) is closely related to the development of RA (27, 28). Mitochondrial peptides can activate the adaptive immune response through the presentation of MHC molecules, while imbalance in mitochondrial homeostasis induces mtDNA mutations and damage, causing peptide sequence changes that can mediate immune cell activation (29, 30). Citrullination of proteins induces the production of ACPAs, which are important biomarkers of RA. Neutrophil extracellular traps (NETs) are a potential source of citrullination antigens. Apart from mitochondrial injury, mitochondrial N-formyl peptides (mtNFPs) concentrations in the plasma of RA patients are elevated and induce enhanced NETs activity in neutrophils *via* the formyl peptide receptor 1 (FPR1)-dependent pathway, promoting the immune response of neutrophils and being an important link in the pathogenesis of RA (31).

## Mitochondrial homeostasis and RA

**Figure 1:**
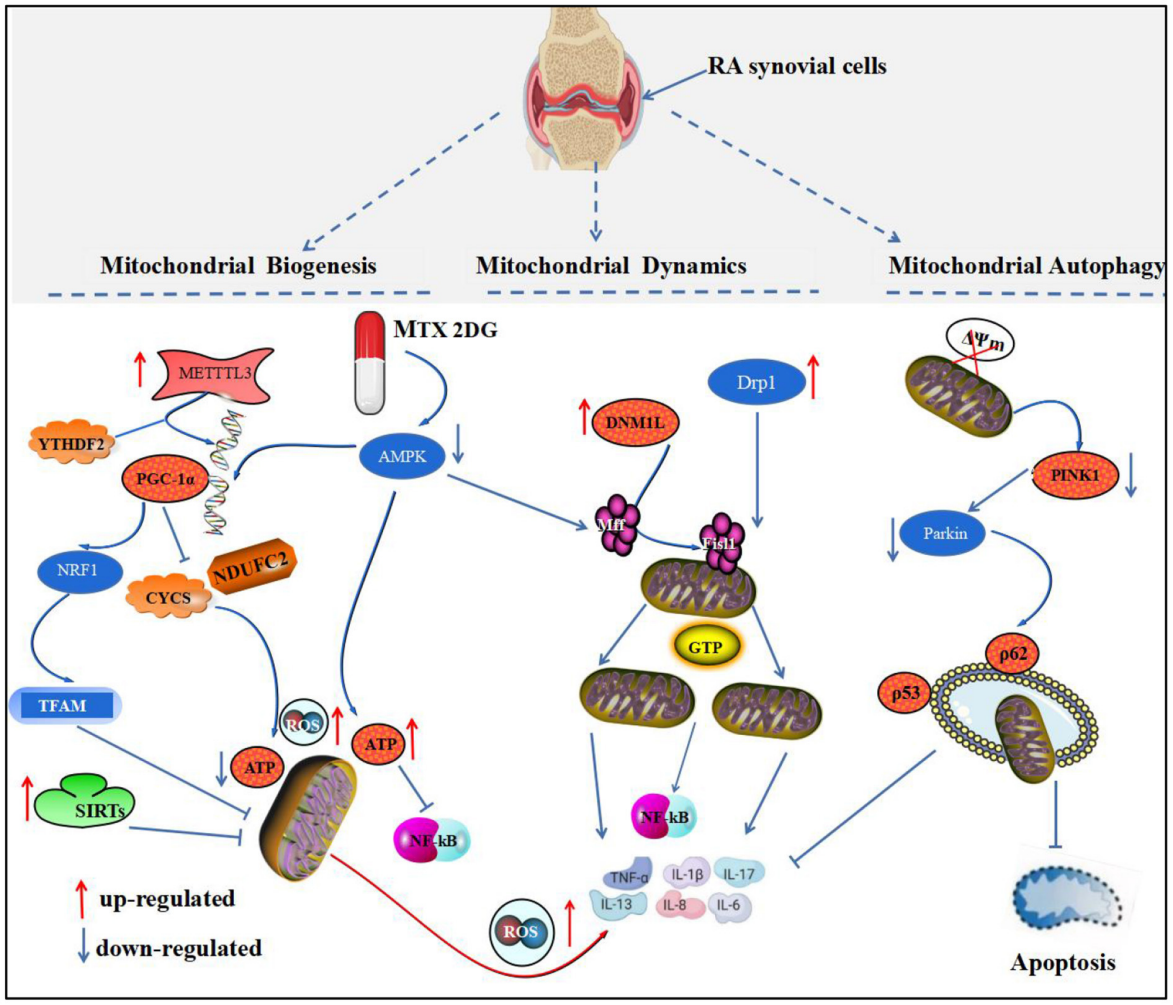
Mitochondrial homeostatic biological processes and the development of RA. This figure depicts the relationship between mitochondrial biogenesis, mitochondrial dynamics, and mitochondrial autophagy and RA. PGC-1 protein is a key protein regulating mitochondrial biogenesis. In RA, PGC-1 mRNA is regulated by METTL3 and YTHDF, which activate the PGC-1α/NRF1/TFAM pathway to inhibit mitochondrial biogenesis and mediate synovial inflammation. Drp1 and DNM1L are key proteins that regulate mitochondrial division, and their aberrant activation in RA binds to the receptors, Mff and Fisl1, and localizes to the outer mitochondrial membrane, mediating mitochondrial fission and the release of inflammatory substances by GTP hydrolase. Disruption of PINK1 and Parkin protein recruitment activates mitochondrial autophagy to inhibit synovial apoptosis and inflammatory factors.

Mitochondrial homeostasis is essential for maintaining the stability of the intracellular environment of synovial cells. RA synovial cells respond to oxidative stress and abnormal energy metabolic changes by altering the structure, number, and function of mitochondria (32). Therefore, we need to interpret the pathogenesis of RA from the perspective of biological processes that maintain mitochondrial homeostasis, which is most closely related to three aspects of mitochondrial biogenesis, altered mitochondrial dynamics, and mitochondrial autophagy (Figure 1).

### Mitochondrial biogenesis and RA

Mitochondrial biogenesis refers to the process of *de novo* mitochondrial formation, which consists mainly of the interaction between nuclear genes and mtDNA. This interaction regulates the self-renewal of organelles and increases the mass of mitochondria in the cell, thus ensuring the stability of the intracellular environment. Mitochondrial biogenesis is coordinated by mtDNA, nuclear DNA (nDNA), and mitochondrial regulatory factors. mtDNA destruction and nDNA degradation leading to impaired mitochondrial biogenesis can mediate the development of chronic inflammatory diseases, such as pulmonary fibrosis, atherosclerosis, and RA (33, 34). Peroxisome proliferator-activated receptor-γ coactlvator-1α (PGC-1α) is a major regulatory protein of mitochondrial biogenesis. It regulates mitochondrial transcription factor A (TFAM), nuclear respiratory factor (NRF), and MTOXPHO genes, which in turn affect mitochondrial biogenesis (35). Additionally, signaling molecules, such as 5'-AMP activated protein kinase (AMPK) and silent information regulator (SIRT), are involved in mitochondrial biogenesis (36).

Methyltransferase-like 3 (METTL3), an RNA N6-methyladenosine (m6A) methyltransferase, is involved in immune and inflammatory regulation. Studies have shown that METTL3 mediates inflammatory responses in AIA rats by activating the NF-κB pathway and can promote FLS activation (37). Additionally, METTL3 and YTHN 6-methyladenosine RNA binding protein 2 (YTHDF2) synergistically mediate the degradation of PGC-1 mRNA and inhibit cytochrome c (CYCS) and ubiquinone oxidoreductase subunit C2 (NDUFC2) gene transcription, thereby decreasing mitochondrial ATP production and oxygen consumption rate (OCR) and increasing mitochondrial ROS accumulation and pro-inflammatory cytokine level in inflammatory monocytes. This study provides insight into the role of m6A methyltransferase METTL3-dependent PGC-1α in monocyte inflammatory response and role of PGC-1α in the pathogenesis of RA from the perspective of PGC-1α-regulated mitochondrial biogenesis (38). PGC-1α is a key protein that mediates mitochondrial biogenesis. It is proportional to the number of mitochondria, and blunting PGC-1α protein leads to reduced mitochondrial gene expression and decreased mitochondrial function, resulting in tissue cell energy metabolism (39). Research (40) showed that quercetin decreased paw clinical arthritis scores and left ankle thickness in CIA mice; reduced inflammatory hyperplasia and cartilage destruction in ankle synovium; and decreased secretion of cytokines, such as TNF-α, IL-1β, and IL-6, by a mechanism similar to that of regulating PGC-1α/NRF1/TFAM pathway activity, promoting mitochondrial biogenesis and ameliorating mitochondrial dysfunction. Conversely, inhibition of PGC-1α mediates atrial energy metabolism disorders and induces apoptosis and fibrosis in atrial tissue cells in CIA rat models, whereas resveratrol corrects metabolic disorders in CIA rat models by activating PGC-1α expression (41).

AMPK is a key protein that senses energy changes and coordinates mitochondrial energy metabolism. Under physiological conditions, activation of AMPK provides ATP for mitochondrial biosynthesis (42). AMPK can directly activate PGC-1α expression to induce mitochondrial neogenesis or catalyze PGC-1α deacetylation by stimulating SIRT1 signaling, which in turn promotes mtDNA replication in mitochondria, which indirectly mediates mitochondrial neogenesis (43). Concurrently, AMPK is a strong inhibitor of ROS and can reduce mtROS production and mtDNA oxidative damage in mediating mitochondrial neogenesis, which in turn inhibits inflammatory response (44). AMPK has been demonstrated to be significantly downregulated in T cells and peripheral blood mononuclear cells in organisms with RA (45), whereas MTX attenuates the production of pro-inflammatory cytokines, such as TNF-α, by activating AMPK (46). The proliferation of synovial cells and secretion of inflammatory substances in RA leads to increased oxygen demand in the joint cavity, and synovial cells shift from phosphorylation to glycolytic pathways to maintain energy supply. The glycolysis inhibitor, 2-deoxy-D-arabino-hexose (2-DG), was found to attenuate inflammatory response in the synovial membrane of AA rats, and the mechanism may be through activation of AMPK to increase AMP or ATP release, improve intracellular metabolism and energy homeostasis to inhibit NF-κB activity, and attenuate synovial cell proliferation and migration in rat models (47). This study provides important evidence for the involvement of energy metabolism in RA synovial inflammation. Similarly, tofacitinib, a newly developed Janus kinase (JAK) inhibitor for RA treatment, significantly reduces the expression of key glycolytic enzyme-related gene enzymes in synovial cells of patients with RA, decreases mitochondrial membrane potential, and increases oxidative phosphorylation energy metabolism to treat RA (48).

SIRTs are a class of nicotinamide adenine dinucleotide (NAD)-dependent histone deacetylases that play an important role in inflammatory diseases. SIRT3, SIRT4, and SIRT5 are located in mitochondria and play an important role in post-translational modifications (PTMs) and activity regulation of mitochondrial proteins (49). SIRT3, SIRT4, and SIRT5 genes and 8-hydroxy-2 deoxyguanosine (8-OHdG) levels were found to be significantly upregulated in synovial tissues of patient with RA, suggesting the key role of SIRTs and oxidative stress in the development of RA (50). Following with SIRT3 knockout, mitochondrial membrane potential and permeability was reduced, function of mitochondrial was impaired in chondrocytes of CIA rat, whereas ryanodine combined with B-cell lymphoma 2 (Bcl-2)-targeted interference siRNA can promote apoptosis in CIA rat fibroblast-like synoviocytes. The underlying molecular mechanism may be related to the down-regulation of Bcl-2 expression and STAT3 (51). SIRT4 gene plays an important role in maintaining the mitochondrial redox state (52), and its high expression promotes TNF-a and IL-6 secretion and accelerates the process of bone destruction in patients with osteoarthritis (52, 53). SIRT5 gene expression is downregulated in the macrophage activation zone, and knockdown of SIRT5 in model rats significantly enhances the release of TNF-a and IL-1β, mediating the development of RA (54). Whereas, SIRT5 reprograms the metabolic process of macrophages to inhibit pro-inflammation by activating PKM2 kinase activity and blocking IL-1β production in macrophages (55). In addition, among the risk factors of lymphoma, the severity of RA is thought to be closely related (56). The overexpression of Sirt1 in diffuse large B-cell lymphoma (DLBCL) leads to the inhibition of PGC-1α protein expression, which affects mitochondrial synthesis (57), the PGC-1α/NRF1/TFAM pathway is inhibited and mitochondrial biogenesis is impaired in RA, which can explains the reasion why RA patients are more susceptible to lymphoma in some degree (40).

### Mitochondrial dynamics and RA

Mitochondrial dynamics is a dynamic equilibrium process in which mitochondria are remodeled by continuous fission and fusion to maintain their normal number, morphology, and function. Dynamin 1-like protein (DNM1L), known as dynamin-related protein-1 (Drp1), is a major regulator of mitochondrial fission, which is recruited by the receptor protein. Mitochondrial fission1 (Fis1)/mitochondrial fission factor (Mff) is recruited to the fission site of the outer mitochondrial membrane, forming an oligomeric ring, which is driven by GTP hydrolase and gradually contracted to cause mitochondrial breakage. Mitochondrial fission allows timely removal of damaged mitochondria, but excessive fission prevents mitochondrial elongation, leading to prolongation or arrest of the cell cycle (58). Mitochondrial fusion is mediated by mitochondrial fusion protein 1 (mitofusin 1, Mfn1) and mitochondrial fusion protein 2 (mitofusin 2, Mfn2). Mfn1 and Mfn2 are anchored to the outer mitochondrial membrane first by their carboxyl termini (hydrolytic guanosine triphosphatase mediates the fusion of the outer mitochondrial membrane), followed by optic atrophy protein 1 (OPA1), which mediates the fusion of the inner mitochondrial membrane (59).

Wang et al. (60) found that DNM1L expression was upregulated in synovial cells of patients with RA, promoting mitochondrial division; whereas DNM1L deficiency induced mitochondrial depolarization and increased synovial cell apoptosis to reduce synovial inflammation. The use of DNM1L inhibitor, mdivi-1, downregulated the AKT/IKK pathway to attenuate NF-κB activation in RA-FLS, inflammatory cytokines, and ROS production, which in turn attenuated the inflammatory response in CIA mice. Therefore, inhibition of DNM1L-mediated mitochondrial division may be a new strategy for the treatment of RA. AMPK directly regulates key mitochondrial fission factors, phosphorylating Mff by binding to serines at sites 155 and 173. Mff phosphorylation recruits Drp1 to bind to its outer mitochondrial membrane, which is the start of the mitochondrial fission process (61). In contrast, after the intervention of the active ingredients, ursolic acid and paeoniflorin from *Cornus officinalis* and *Paeonia lactiflora*, respectively, the ratio of MTFP1 expression and p-Drp1 (Ser616/Ser637) in the synovial tissue of CIA rat models was significantly reduced. The expression of Mfn2 was, however, upregulated, enhancing the expression of the pro-apoptotic proteins, Bax, caspase-3, and cytochrome C, in the synovial tissue of CIA rats, suggesting that it can promote mitochondrial fission and inhibition of mitochondrial fusion to induce apoptosis in synovial cells. The mechanism may be related to the activation of AMPK signaling pathway by mitochondrial fission (62).

### Mitochondrial autophagy and RA

Mitochondrial autophagy is a form of autophagy that maintains the integrity of mitochondrial structure and function and cellular homeostasis by degrading and removing dysfunctional mitochondria from the cytoplasm. Tensin homologous inducible kinase 1 (PINK1) and PARK2-mediated mitochondrial autophagy is a widely studied pathway (63). Disruption of the mitochondrial transmembrane potential (ΔΨm) leads to the activation and localization of PINK1 in the outer mitochondrial membrane (OMM) and the subsequent recruitment and activation of Parkin by PINK1, which further selectively recruits autophagy receptors, such as nuclear pore protein 62/isolated vesicle 1 (p62/SQSTM1) and optineurin/optin, thereby initiating mitochondrial autophagy (64).

Mitochondrial autophagy levels were significantly reduced in patients with RA (65), and PINK1-mediated mitochondrial autophagy was inhibited in an inflammatory setting (66). Parkin expression was significantly downregulated, whereas Parkin reduced the inflammatory response to arthritis by inhibiting p53 degradation (67). miR-144-3p, through the PINK1/Parkin axis, promotes IL-1β-induced chondrocyte scorching (68). RA-associated lung disease (RA-ILD) is one of the more serious complications of RA with high mortality. Its pathogenesis is related to genetic, immune and other factors. The pathogenesis is mainly abnormal tissue reaction in alveolar wall and lung parenchyma (69). It was found that the expression of Unc-51 like autophagy activated kinase 1 (ULK1), p62Beclin-1, E3 ubiquitin ligase (PRKN) and PINK1 protein were up-regulated in RA-ILD patients (65). It is indicated that mitochondrial autophagy may be involved in the occurrence of RA-ILD. In the study of ILD, the deficiency of mtDNA, the decrease of respiratory activity and the increase of ROS production are closely related to the pathogenesis (70). In FLS of patients with RA and AIA rat models, PTEN was downregulated and DNA methyltransferase (DNMT)-1 was upregulated. Intervention with TNF-α inhibitors resulted in increased DNA methylation of PTEN, increased autophagic activity in synovial tissue, and foot and plantar swelling relief in AIA rats, whereas the methylation inhibitor, 5-azacytidine, inhibited the release of cytokines and chemokines and the activation of FLS *in vitro*. These studies provided therapeutic strategies for PINK1- and PARK2-mediated mitochondrial autophagic pathways for the treatment of RA (71).

## Mitochondrial homeostasis and energy metabolism of immune cells

Innate immune cells, such as T cells, macrophages, and dendritic cells (DCs), play an important role in mediating the immune inflammatory response in RA (72). Abnormal activation of these cells may lead to excessive production of pro-inflammatory cytokines, such as TNF-α, IL-6, and IL-17 (73, 74). The energy required for the survival and biological behavior of immune cells is mainly derived from glucose, fatty acids, and amino acids, which are involved in the tricarboxylic acid cycle through glucose metabolism (glycolysis), fatty acid β-oxidation (FAO), and glutaminolysis, respectively. Mitochondria are the energy metabolism production sites of immune cells, and the homeostatic environment provides for the production of ATP mediated by mitochondrial oxidative phosphorylation (OXPHOS). Studies have confirmed that disturbance of immune cell metabolism in synovial tissues is a key factor in the development of RA. Since the inflammatory synovial cavity of patients with RA is a relatively hypoxic local microenvironment, abnormal alterations in energy lead to abnormal activation of various cells, including CD4^+^T-cell subsets and DC cells, which participate in the pathological process of RA. Alterations in energy metabolism are closely related to imbalances in mitochondrial homeostasis (75, 76). Therefore, we need to discuss mitochondrial homeostasis and the generation, effects, and regression of immune response in RA, in terms of different immune cell energy metabolic pathways, such as glucose, fatty acid β-oxidation, and glutamine metabolism.

### T-cell subsets

T cells bind specifically to antibodies *in vivo* and extensively mediate the immune inflammatory response. Th17 cells produce various pro-inflammatory cytokines to promote synovial inflammation, whereas Treg cells suppress inflammation to maintain immune tolerance (77, 78).

Glycolysis is the main pathway of T-cell glucose metabolism, producing lactate under anaerobic conditions and carboxylating under aerobic conditions to produce acetyl coenzyme A (CoA), which enters the mitochondrial triphosphate cycle to produce ATP. Studies have shown that glycolysis drives mitochondrial division and increases the release of IL-1β (79). The expression of HK2, fructose-6-phosphate 2-kinase/fructose-2,6-bisphosphatase 3 (PFKFB3), and pyruvate kinase 2 (PKM2), all metabolism-related enzymes of the glycolytic pathway, were significantly increased in RA-FLS, suggesting increased aerobic glycolysis in RA-FLS (80, 81). In RA CD4^+^ T cell metabolism, glucose is catabolized to ATP by the glycolytic pathway. The catabolic process is defined as a danger-associated molecular pattern (DAMP) due to reduced biological stability of mitochondrial DNA, which is caused by the lack of the DNA repair nuclease (MRE11A). Additionally, it is caused by increased oxidative damage to mtDNA and increased permeability of the RA environment, due to the lack of N-myristoyltransferase 1 (NMT1). Leakage into the cytoplasm initiates the assembly of inflammatory vesicles and activates cystein-1, releasing IL-1β and IL-18 (82, 83). Meanwhile, in the physiological state, mitochondria produce ATP for cellular energy through oxidative phosphorylation, and the imbalance in homeostatic environment leads to impaired oxidative phosphorylation metabolism, resulting in mitochondrial oxidative stress and exacerbating damage to mitochondrial proteins, nucleic acids, and lipids (84). Contrarily, in autoimmune diseases, CD4^+^ T differentiation to Th17 cells, increased mitochondrial fission and suppressed levels of oxidative phosphorylation activate mTOR/STAT3/HIF-1 expression and promote the glycolytic process (85).

**Figure 2:**
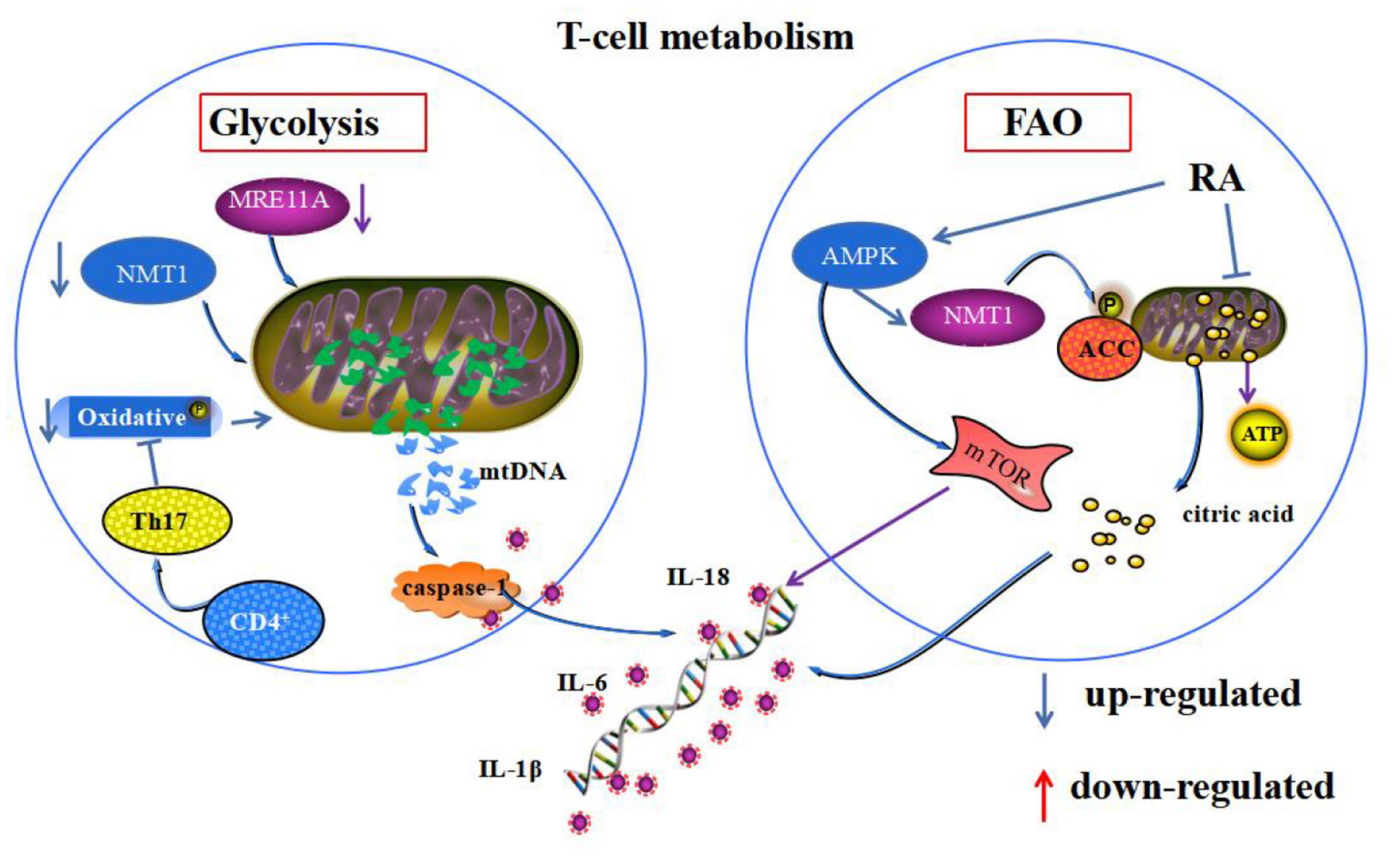
Mitochondrial homeostasis and T-cell energy metabolism. Both glycolysis and FAO mediate T-cell energy metabolism. Deficiency of MRE11A and NMT1 and oxidative phosphorylation during glycolysis metabolism in RA synoviocytes promote the leakage of mtDNA from mitochondria, leading to the secretion of inflammatory substances. During FAO in RA synoviocytes, abnormal activation of AMPK phosphorylation of ACC leads to mitochondrial citric acid transport to the cytoplasm, mediating the secretion of inflammatory substances. Additionally, it activates mTOR, which inhibits mitochondrial autophagy.

FAO is an alternative pathway for regulatory T cells to provide energy to the cell through mitochondrial oxidative metabolism of lipids during emergencies, such as infection and starvation. β-oxidative phosphorylation of mitochondria in RA T cells is reduced, and oxidative emergencies are increased. These changes disrupt the mitochondrial homeostatic environment and accumulate fatty acids in the cytoplasm (86). AMPK is an AMP-dependent protein kinase, with the N-myristoyl base transferase 1 (NMT1), placed on the cytoplasmic surface of lysosomes to sense intracellular energy changes (87). Its activation phosphorylates acetyl coenzyme A carboxylase (ACC), promoting intracellular fatty acid β-oxidation and inhibiting lipid synthesis. Meanwhile, impaired production of succinate, a mediator of mitochondrial production in RA CD4^+^T cells, which leads to impaired mitochondrial biogenesis and inability to convert acetyl coenzyme A to ATP, generates excess citrate. The excess citrate is transported from mitochondria to the cytoplasm, inducing differentiation of pro-inflammatory effector cells and cytokine and inflammatory factor release (88). Activation of AMPK-related pathways may promote regulatory T cell differentiation and thus anti-inflammatory and therapeutic effects in RA (89). However, whether this is achieved based on the correction of homeostatic imbalance by AMPK regulation of fatty acid β-oxidation in mitochondria still needs further investigation. In patients with RA with CD4^+^T cells, mitochondrial dysfunction, reduced glycolysis, and less pyruvate and ATP production in T cells resulted in activation of AMPK. Whereas, impaired NMT1 enzyme function and impaired post-translational lipidation modification of proteins activated mTORC1 expression, induced fatty acid β-oxidation, promoted synovial inflammation by inhibiting mitochondrial autophagy, and suppressed catabolism (90) (Figure 2).

### Macrophages

Macrophages are important effector cells in the development of RA. They interact with various cells, such as T and B cells and fibroblast-like synoviocytes, to produce large amounts of inflammatory cytokines, leading to pannus formation and destruction of bone and cartilage (91). Increased M1 macrophages in RA joints requires more energy to perform their functions, leading to a shift in glucose metabolism from oxidative phosphorylation to glycolysis involved in the pathological process of RA. Additionally, fatty acid oxidation mediates the involvement of M2 macrophages in the TCA cycle, which is essential for the pathogenesis of RA (92).

The presence of large amounts of lactate and TCA cycle intermediate metabolites in RA synovial fluid, reduced levels of oxidative phosphorylation (OXPHOS), and active glycolytic metabolism (93) generates pyruvate molecules that are converted to acetyl coenzyme A in the mitochondria. Acetyl coenzyme A enters the TCA cycle and eventually generates ATP. After LPS stimulation, macrophages of synovial membrane adjust from the mitochondrial TCA cycle pathway to a succinate-dependent pathway. Thereafter, glycolysis assumes the role of ATP production, enabling mitochondria to maintain high membrane potential, inducing mitochondrial ROS production and driving IL-1β production (94). Hypoxia and HIF-1-activated glucose transporter-1 (GLUT1) can increase glucose uptake and glycolysis flux of macrophages, leading to mitochondrial stress and ROS production and release of TNF- α and IL-1 β. These changes mediate synovitis and cartilage and bone damage (95, 96). The mitochondrial autophagy receptor BNIP3L/NIX-dependent mitochondrial autophagy manipulates metabolic reprogramming toward glycolysis, supporting inflammatory macrophage polarization to generate a rapid immune response during inflammation (97). Activating M1 macrophages and reducing OXPHOS expression through ROS inhibitors help to improve M1 macrophage repolarization into M2 macrophage repolarization. They can be effective ways to control inflammatory diseases (98).

FAO mediates the involvement of macrophages in the TCA cycle with increased fatty acid synthesis in M1 macrophages and enhanced FA uptake and FAO in M2 macrophages in RA (99). Studies have shown that IL-4 and IL-13 increase macrophage peroxisome proliferator-activated receptor γ coactivator 1β (PCG-1β) expression, leading to increased mitochondrial respiratory chain protein expression and accelerated FAO (73, 100). In turn, this can lead to cholesterol accumulation from acetyl coenzyme A production, thereby increasing the risk of atherosclerosis in RA. Activated macrophages in RA have a special affinity for choline. Choline uptake is enhanced, and choline kinase (Chok) phosphorylates choline to produce phospholipids. Thereafter, macrophages are induced to produce inflammatory cytokines (101). Inhibition of choline or Chok leads to mitochondrial damage, decreased ATP synthase activity and intracellular ATP, and subsequent activation of adenylate-activated protein kinase, which promotes macrophage mitosis and inhibits IL-1β production by macrophages (102).

Concurrently, pro-inflammatory macrophages exhibit two disruptions in the TCA cycle, leading to the accumulation of citrate and succinate and the induction of an arginine-succinate shunt (AST) to support NO production (103). Arginine is an important amino acid for RA macrophage metabolism; it is a substrate for arginase (Arg) and nitric oxide synthase (NOS) (104). Arginase 2 (Arg2) is a miR-155 and IL-10 regulatory protein, and Arg2 deficiency in LPS-stimulated macrophages leads to increased secretion of mtROS, HIF-1α, and IL-1β. Arg2 increases succinate dehydrogenase/complex II activity and contributes to increased mitochondrial fission (105), showing changes, such as fragmented morphology, loosened mitochondrial cristae, and decreased metabolic program. Concurrently, increased mitochondrial production of ROS drives NF-κB-dependent transcription of inflammatory cytokines (106).

## Mitochondrial homeostasis and synovial cell apoptosis and proliferation

Both apoptosis and proliferation are indispensable cellular physiological processes. They are essential for maintaining the dynamic balance of cell numbers *in vivo*. Imbalance between apoptosis-inhibiting and apoptosis-promoting factors in RA synovial cells leads to prolonged lifespan of synovial cells, thickening of synovial lining layer, release of inflammatory secretory substances, and increased secretion of matrix metalloproteinases, promoting synovial inflammation and cartilage and bone tissue damage. During the development of RA, alterations in the mitochondrial homeostatic environment in RA-FLS, mainly including abnormalities in fission and autophagic activity, are involved in their synovial cell metabolism and apoptosis (107). These do not only secrete inflammatory cytokines and chemokines to promote inflammatory responses but also promote matrix metalloproteinase secretion, mediating chondrocyte degradation, stimulating osteoclast differentiation, and leading to bone erosion (108).

DNM1L is a key regulator of mitochondrial division. Silencing DNM1L expression increases mitochondrial length in FLS and induces mitochondrial depolarization. Inhibition of mitochondrial fission decreases the ratio of LC3B-II to LC3B-I in FLS, inducing FLS apoptosis to reduce inflammation in RA synovial cells (59, 109). The accumulation of the pro-inflammatory mediator, AOPP, in patients with RA triggers the leakage of mtROS, which in turn increases the content of Bax and cytochrome C in the chondrocytoplasm and decreases the content of the anti-apoptotic protein, Bcl-2 (110). miR-125b expression is reduced in peripheral blood mononuclear cells of patients with RA, which can induce apoptosis by negatively regulating the pro-apoptotic protein, BIK/mitochondrial fission process 1 protein (MTP18), leading to reduced oxidative phosphorylation and mitochondrial fission (111). Another study found that FLS in patients with synovitis exhibited significantly elevated mitochondrial respiratory capacity in the resting state. They were less fragmented in patients with synovitis than in patients with early persistent RA (veRA) in reduced morphological fragmentation after mitochondrial division, following TNF-α inhibitor treatment (112).

Increased ROS production in RA-FLS mediated by tumor necrosis factor-like ligand 1A (TL1A) induces activation of the NF-κB/STAT3 pathway and increases the levels of IL-6 and IL-8 (113). Concurrently, the inflammatory environment induces the accumulation of ROS and leads to mitochondrial dysfunction in synovial cells, further increasing the expression of the key proteins of autophagy, ATG5 and LC3, and autophagic vesicles, which by inhibiting autophagy can mediate the anti-apoptotic effects of cells (114). Resveratrol does not only reduce the expression of oxidative stress-related proteins, SIRT3 and MnSOD, to enhance mtROS production but also inhibits FLS proliferation and induces FLS apoptosis, which are related to the ability of resveratrol to reduce expression of autophagy proteins, Beclin1 and LC3A/B (115, 116).

**Figure 3:**
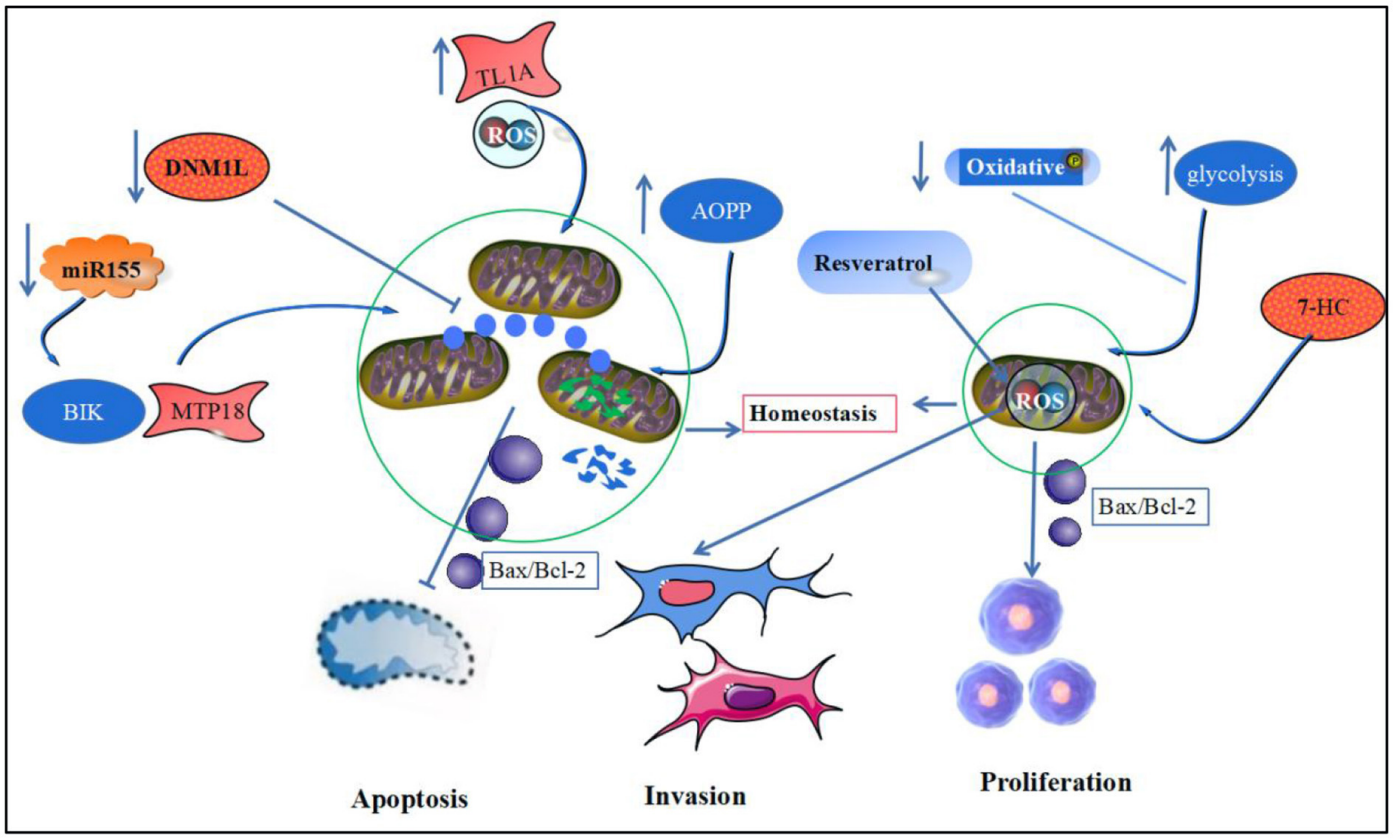
Mitochondrial homeostasis and RA synoviocyte apoptosis and proliferation. This figure depicts mitochondrial homeostasis and synoviocyte apoptosis, proliferation, and invasion. Downregulation of DNM1L, AOPP, and miR-125-b expression and upregulation of TL1A expression in RA led to disruption of the mitochondrial homeostatic environment and increased release of the anti-apoptotic protein, Bax/Bcl-2, promoting the onset of synovial cell apoptosis. Meanwhile, oxidative phosphorylation in RA synoviocytes was weakened, and glycolysis was increased, forcing increased release of ATP from mitochondria and leading to increased proliferation and invasive activity of synoviocytes.

The invasion and proliferation of synovial cells is another major pathological change in RA, whereas apoptosis mediated by the mitochondrial pathway can control synovial cell proliferation and pannus formation in RA (117). Oxidative stress inducers were found to reprogram RA fibroblastic synovial cell metabolism by downregulating mitochondrial oxidative phosphorylation and promoting glycolysis, which in turn promotes synovial lining cell proliferation and pannus formation (118). Meanwhile, in a severely hypoxic environment, RA synovial tissue induces HIF-1α and NF-κB expression by downregulating mitochondrial respiration, increasing glycolysis and lactate metabolism, which in turn mediates abnormal angiogenesis, cell proliferation invasion and vascular pannus formation (119). Studies have shown that epimedoside, the active ingredient of the traditional Chinese medicine, Epimedium, inhibits the migration and proliferation of FLS in a concentration-dependent manner and induces RA-FLS apoptosis by reducing the mitochondrial membrane potential and enhancing the mitochondrial pathway-related apoptotic proteins in FLS (120). Additionally, 7-hydroxycoumarin (7-HC) can inhibit FLS lineage cell (MH7A) proliferation by inhibiting the Wnt/β-catenin pathway or modulating mitochondrial-mediated Bax/Bcl-2 activity and induce FLS apoptosis (121). Another study showed that resveratrol could inhibit mtROS production by activating the Nrf2 pathway, thereby inhibiting the activation of NF-κB and proliferation, migration of RA-FLS (122). β-elemene from Chinese medicinal herb, *Curcuma wenyujin* extract, induced MAPK phosphorylation, which in turn activated in FLS caspase-3, caspase-9, and cytochrome C expression, contributes to apoptosis in synovial cells (123) (Figure 3).

## Mitochondrial homeostasis and inflammatory pathways

**Figure 4:**
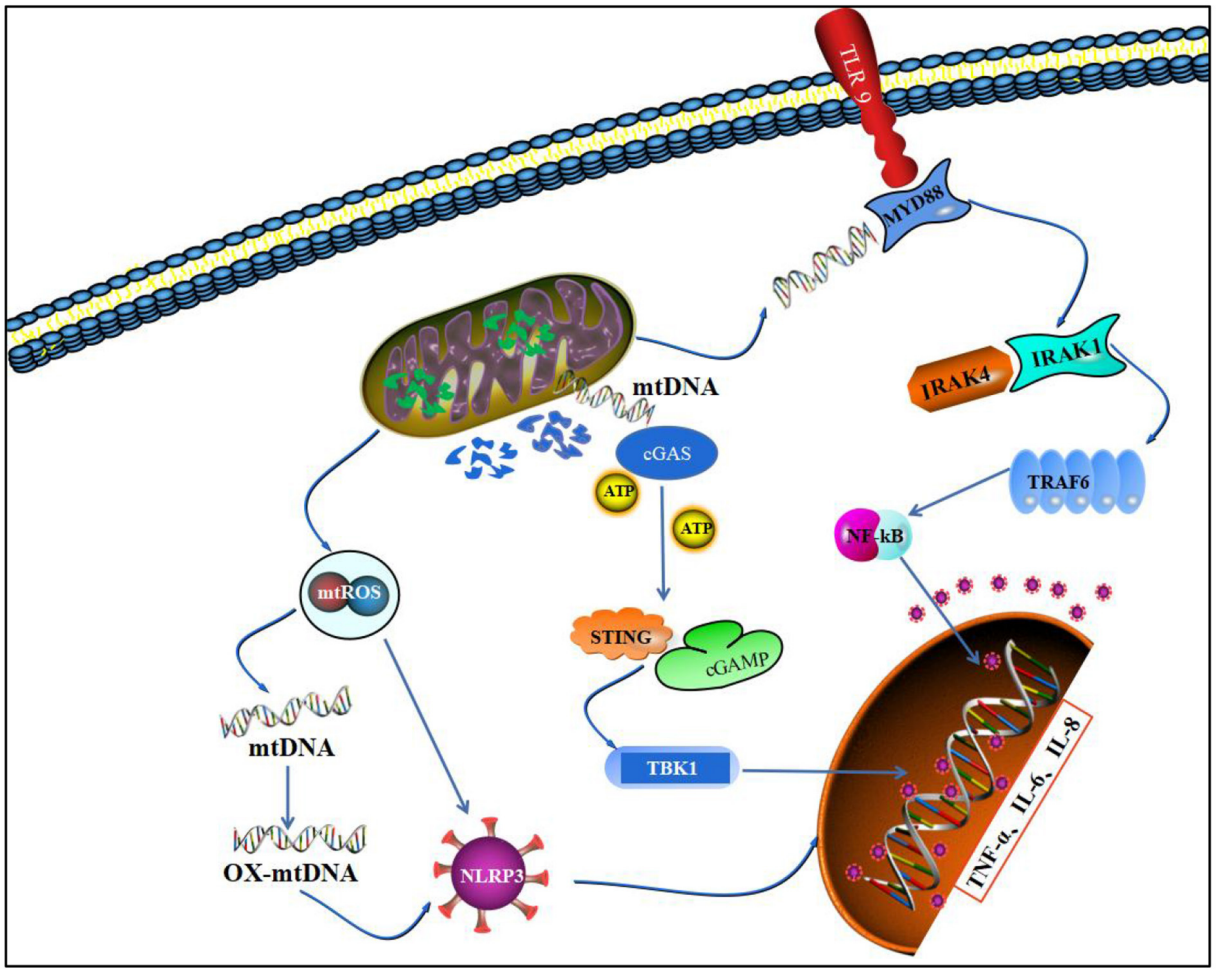
Mitochondrial homeostasis and inflammatory pathways. This figure depicts that imbalance in mitochondrial homeostasis leads to leakage of mtROS and degradation of mtDNA and promotes nuclear transcription of inflammatory factors through three pathways, NLRP3, TLR9/NF-κB, and cGAS/STING.

The pathological process of RA involves extensive infiltration of inflammatory cells, such as macrophages, lymphocytes, and neutrophils, and massive release of inflammatory cytokines, such as TNF-α, IL-1β, and IL-6. Furthermore, it causes a persistent synovial inflammatory response through a series of cascade reactions (124). Inflammatory cells produce large amounts of inflammatory secretions associated with a sustained inflammatory response by autocrine or heterocrine means, leading to joint swelling and interaction with inflammatory cytokines to form a complex network system (125). Although mitochondria are commonly thought to be associated with energy production, studies have shown that they can coordinate the immune inflammatory response through multiple pathways and play an important role in linking acute joint tissue injury, inflammation, and long-term chronic joint degeneration (126) (Figure 4).

### Mitochondrial homeostasis and the assembly and activation process of NLRP3

Studies have shown that NLRP3 is a multi-protein complex that responds to pathogenic microorganisms and directly responds to DAMPs released within the joint after injury (127). When NLRP3 accepts an activation signal, it activates caspase-1, enabling it to cleave IL-1β into its active form, and the whole process is controlled by mitochondria. Specifically, mitochondrial membrane potential, mitochondrial ROS, and mtDNA are jointly involved in the activation of NLRP3 inflammatory vesicles (128). Jian (129) demonstrated that LPS-induced macrophage inflammation and NLRP3 activation require mtROS involvement, and the application of mitochondria-targeted antioxidants can inhibit NLRP3 activation and reduce IL-1β production (130). Additionally, a large amount of mtROS will lead to the generation of oxidative-mitochondrial genes (ox-mtDNA) by peroxidative damage to mtDNA. Oxidatively damaged bases in ox-mtDNA have more affinity to NLRP3 and are a key pathway for NLRP3 initiation. NLRP3 activation can cause uncontrolled inflammatory responses, and NLRP3 is involved in the onset and development of RA (131). Meanwhile, cell scorch death, a specific form of inflammatory cell death, is closely associated with abnormal synovial proliferation and osteochondral destruction in RA. It is regulated by mitochondrial homeostasis, and cell scorch death activates NLRP3, leading to the release of IL-1β and DAMPs, which enhances the inflammatory response (132).

### Mitochondrial homeostasis and TLR9/NF-κB

Mitochondrial outer membrane damage predisposes contents, such as mtDNA, to leak into the cytosol, and the hypomethylated CpG sequence in mtDNA can bind specifically to the N-terminal end of the C-shaped leucine-rich repeat region of TLR9, mediating TLR9 activation (133, 134). TLR9 activation mediates NF-κB phosphorylation through the MyD88 pathway, which in turn nuclear translocates and transcribes IL-1β, IL-6, TNF-α, GM-CSF, and many other inflammatory factors, which are released and form key part of the mitochondrial injury-mediated inflammatory response. mtDNA injection into the joint cavity of mice can promote inflammatory arthritis in mice, whereas nuclear DNA injection has no similar effect, thus validating the inflammatory properties of mtDNA. The pathological process of mtDNA may be related to the ability of myeloid cells to regulate NF-κB activity. Synthetic oligodeoxynucleotides containing oxidized residues are able to induce inflammatory pathology, whereas non-oxidized forms of the same sequence have no inflammatory effect, suggesting that oxidized DNA have greater immunostimulatory properties (135).

Damaged mitochondrial DAMPs (MTDs), which include formyl peptide and mitochondrial DNA, enter the immune cycle sequence and activate human polymorphonuclear neutrophils (PMNs) *via* formyl peptide receptor-1 and TLR9, respectively. MTDs promote PMN Ca^2+^ flux and mitogen-activated protein (MAP) kinase phosphorylation, leading to PMN migration and degranulation *in vitro* and *in vivo*. Migration, degranulation, and these signals are activated *via* innate immune pathways identical to those activated in sepsis to produce a sepsis-like state, and the release of these mitochondrial “internal enemies” through cellular injury is a key link between trauma and immune inflammation (136).

### Mitochondrial homeostasis and the cGAS/STING pathway

The cyclic GMP-AMP synthase (cGAS)-stimulator of interferon gene (STING) signaling plays an important regulatory function in microbial and cellular immunity through the induction of type I interferons, including cytokines. mtDNA release into organelles after massive damage can be cGAS/STING pathway sensing. It interferes with basic intracellular homeostatic processes (apoptosis, autophagy) and regulates cellular metabolism (137). mtDNA leakage can mediate activation of the cGAS/STING pathway in the cytosol. mtDNA can bind to cyclic guanosine adenylate synthase (cGAS), prompting cGAS to form cyclic GMP-AMP with GTP and ATP (cGAMP). cGAMP further activates interferon gene stimulating factor (STING) and promotes ANK-binding kinase 1 (TBK1)-mediated phosphorylation of interferon transcription factor 3 (IRF3) (138), and the activation of IRF3 in the nucleus transcribes various inflammatory factors, such as IL-1β, TNF-α, and CCL2 (139). Additionally, the activated STING/IRF3 pathway activates NLRP3 and NF-κB pathways, which play key roles in the inflammatory response network (140).

## Future direction

Mitochondrial homeostasis is critical in maintaining energy metabolism in RA. Regulation of mitochondrial homeostasis is an effective target for the treatment of RA. Therefore, targeting pathways of energy metabolism in a homeostatic environment can treat various diseases, including cardiovascular, neurodegenerative, and autoimmune diseases (141). Excellent treatment results are achieved by modulating mitochondrial homeostasis from many of the current targeted therapies for RA. Most frontier anti-rheumatic drugs commonly used clinically maintain mitochondrial homeostasis by directly modulating mitochondrial biogenesis, mitochondrial dynamics, and autophagy. An example is the traditional synthetic detoxification antirheumatic drug (csDMARD), methotrexate (MTX), which can inhibit the mitochondrial folate pathway by competitive inhibition of dihydrofolatereductase (DHFR), affecting mitochondrial biogenesis and increasing anti-proliferative effect (142). Additionally, leflunomide inhibits proliferation by inhibiting the mitochondrial inner membrane protein of dihydroorotate dehydrogenase (DHODH), which reduces the ab initio synthesis of pyrimidines. The depletion of pyrimidines of leflunomide can promote the expression of mitogenic proteins 1 and 2 (Mfn1/2) and increase mitochondrial fusion (143). Sulfasalazine has anti-proliferative and cytotoxic effects; it is an effective treatment for autoimmune diseases. It induces apoptosis of synovial T cells by increasing mitochondrial permeability (144). Meanwhile, the toxicity of DMARDs maybe predicted by mitochondrial activity. HepG2 cells exposed to 31.25 and 62.5 μM of leflunomide and its derivative A771726 for 2 h and found that the levels of mitochondrial ATP was decreased with time and concentration dependent manner (145). The mitochondrial succinate dehydrogenase (SDA) activity was reduced and mitochondrial GSH was depleted after the intervention with salazosulfapyridine (146).

However, many targeted biologic therapies for RA cannot directly target mitochondria. Rather, they block cytokine signaling pathways, especially inflammatory cytokine signaling, which has a powerful effect on mitochondrial biology. For example, when TNF-a blockers are used, the frequencies of mitochondrial fission and markers of oxidative stress are reduced in the synovial membrane of patients with RA by a mechanism that may be related to the inhibition of ROS and nitrogen production (147). 2-DG, a non-competitive inhibitor of HKs, restores mitochondrial homeostatic imbalances by reversing lipopolysaccharide-induced glycolytic activity, thereby inhibiting the expression of RA inflammatory cytokines, IL-1β and IL-6, and matrix metalloproteinase (MMP-1, MMP-3) (148). Similarly, tofacitinib, a newly developed JAK inhibitor for RA treatment, significantly reduces the expression of key glycolytic enzyme-related genes and enzymes in synovial cells of patients with RA, decreases mitochondrial membrane potential, and increases oxidative phosphorylation energy metabolism to treat RA (149) (Table 1).

**Table 1:** Effects of common anti-rheumatic drugs on mitochondrial homeostasis.

**Drugs**	**Action point**	**Effect on mitochondria**	**Impact on RA**	**References**
**Mitochondrial biogenesis**				
MTX	Folate-dependent ↓	Inhibits mitochondrial folate synthesis	Inhibits synovial cell proliferation	(144)
Quercetin	PGC-1α ↓	Promotes mtDNA	Inhibits TNF-α, IL-6, etc.	(39)
Resveratrol	PGC-1α ↓	Promotes mtDNA	Corrects RA metabolic disorders	(40)
2-DG	Glycolysis ↓	Activates AMPK and improves energy metabolism	Inhibits NF-κB	(148)
Tofacitinib	JAK ↓	Reverses mitochondrial membrane potential	Lowers glycolytic enzyme genes	(152)
IRF9	SIRT1 ↑	Reverses mitochondrial membrane potential and increases in membrane permeability	NF-κB inhibition and pro-apoptosis	(151)
Cantleyoside	SIRT1 ↑	Suppresses OCR and ECAR	NF-κB inhibition and pro-apoptosis	(151)
Metformin	ETC complexes ↑	Reduces mitochondrial activity and ATP production	Inhibits synovial cell proliferation	(153)
**Mitochondrial dynamics**				
mdivi-1	DNM1L ↓	Promotes mitochondrial division	Promotes apoptosis of synovial cells	(83)
Leflunomide	DHODH ↓	Inhibits DHODH, promotes Mfn1, Mfn2, and increases mitochondrial fusion	Reduces oxidative stress and inhibits proliferation	(143)
Sulfasalazine		Increases mitochondrial membrane permeability	Induces T-cell apoptosis	(144)
TNF-a inhibitor	TNF-a ↓	Reduces mitochondrial division	Reduces oxidative stress	(109)
Icariside		Reverses mitochondrial membrane potential	Induces RA-FLS apoptosis	(120)
7-HT		Enhances mitochondrial pathway Bax/Bcl-2	Induces apoptosis in FLS cells	(121)
β-Elmene		Phosphorylates MAPK pathway	Induces apoptosis	(123)
**Mitochondrial autophagy**				
5-azacytidine	Methylation ↓	Enhances PINK1-mediated mitochondrial autophagy	Inhibits cytokines and chemokines	(65)
Rapamycin	mTOR ↑	Enhances mitochondrial autophagic activity in synovial cells	Inhibits cytokines	(154)

In conclusion, modulation of PGC-1α protein in mitochondrial biogenesis inhibits inflammatory response in RA, whereas AMPK, a key protein in sensing energy changes, has been widely used in the clinic as an interventional target (150). Sirtuins are a series of protein of histone deacetylases that play an important role in inflammatory diseases, and sirtuin agonists and natural drugs have been previously used in the treatment of RA (49). Mitochondrial fission and fusion undergo a dynamic equilibrium process of remodeling to maintain their normal number, morphology, and function, and inhibition of mitochondrial fission may be a new strategy for the treatment of RA. Mitochondrial autophagy maintains the mitochondrial homeostatic environment. A direct link exists between the identification of the targets of action of mitochondrial autophagy-related proteins and the pathological features of RA. Therefore, elucidation of the mechanisms regulating mitochondrial homeostasis in RA and identification of the precise and appropriate biological processes regulating mitochondrial homeostasis to make the prevention and treatment of RA beneficial are important. Concurrently, the bilayer membrane structure of mitochondria is a key barrier to the action of certain drugs, and more drugs targeting mitochondria or increasing the permeability of drugs to the inner and outer mitochondrial membranes through nanotechnology are needed to translate basic research findings into clinical drug development for the benefit of patients with RA.

## Author contributions

LC wrote the manuscript. DX and WH revised the manuscript. JW conceived the idea for the study. SC, LL, ZX, and YB performed the literature search and review. All authors contributed to the article and approved the submitted version.

## Funding

This work was supported by the National Natural Science Foundation of China (No. 82060891), Natural Science Foundation of Gansu Province (No. 21JR7RA568), Project of Zheng's Acupuncture Academic Schools of Heritage Studio, Gansu Province, State Administration of TCM (No. 2305135901), and Gansu Province Youth Science and Technology Fund (No. 20JR10RA344) funded the study.

## Conflict of interest

The authors declare that the research was conducted in the absence of any commercial or financial relationships that could be construed as a potential conflict of interest.

## Publisher's note

All claims expressed in this article are solely those of the authors and do not necessarily represent those of their affiliated organizations, or those of the publisher, the editors and the reviewers. Any product that may be evaluated in this article, or claim that may be made by its manufacturer, is not guaranteed or endorsed by the publisher.
